# Esophageal metastasis of breast cancer during endocrine therapy for pleural dissemination 21 years after breast surgery: a case report

**DOI:** 10.1186/s40792-019-0585-x

**Published:** 2019-02-15

**Authors:** Masuyo Miyake, Akimitsu Yamada, Kentaro Miyake, Itaru Endo

**Affiliations:** 1 Department of Breast Surgery, Chigasaki Municipal Hospital, Honson 3-5-1, Chigasaki, Kanagawa 253-0042 Japan; 2 Department of Gastroenterological Surgery, Yokohama City University School of Medicine, Fukuura 3-9, Kanazawa, Yokohama, Kanagawa 236-0004 Japan

**Keywords:** Breast cancer, Esophageal metastasis, Late recurrence, Endocrine therapy, Radiation therapy

## Abstract

**Background:**

The esophageal metastasis of breast cancer is rare. Moreover, it is extremely unusual for patients to experience the symptoms of esophageal metastasis during their lifetimes. We present a case of dysphagia caused by esophageal metastasis after a long interval following a primary mastectomy.

**Case presentation:**

A 77-year-old woman with a history of heterochronous bilateral breast cancer and under treatment for pleural dissemination recurrence originating from right breast cancer complained of dysphagia. At the age of 56, she had undergone a right radical mastectomy for right breast cancer. The histopathological findings revealed invasive ductal carcinoma, pT3N1M0, which was estrogen receptor (ER)- and progesterone receptor (PgR)-positive. At the age of 73, she underwent a second operation, a left modified radical mastectomy. The histopathological examination revealed invasive ductal carcinoma, pT1N0M0, which was negative for ER, PgR, and human epidermal growth factor receptor 2 (HER2). Four years after completion of adjuvant therapy for the left breast cancer, pleural effusion on her left side was observed and histopathological examination of a sample revealed pleural dissemination resulting from the right breast cancer. After initiation of therapy for recurrence, she developed dysphagia and, therefore, underwent an upper gastrointestinal tract endoscopic examination. The examination revealed whole circumferential stenosis and a band unstained by Lugol’s solution located 30 cm from her incisors. Examination of a biopsy specimen revealed a subepithelial luminal structure and dysplastic cells. Immunostaining was positive for CK7 and negative for CK20; furthermore, the sample was ER and PgR-positive. Considering the pathological findings, the patient was diagnosed with esophageal metastasis of her right breast cancer.

**Conclusions:**

Metastatic lesions in the esophagus are often located in the submucosa; therefore, they may not be definitively diagnosed by histopathological examination of mucosal biopsy specimens. Esophageal metastasis originating from breast cancer often occurs as a part of multiple organ metastases; however, esophageal metastasis is usually not considered a prognostic factor for patients. Therefore, treatment should be determined according to the severity of the other metastatic sites and the degree of esophageal stenosis.

## Background

Breast cancer has a prolonged course and has the potential for systemic metastasis. The common sites of breast cancer metastasis include the lymph nodes, liver, bone, lung, and brain, while esophageal metastasis is rare. Case reports of esophageal metastasis from breast cancer are mainly autopsied cases; therefore, patients with symptoms of esophageal metastasis who are diagnosed while alive are extremely unusual. We present a case of dysphagia caused by esophageal metastasis 21 years after the patient had undergone a primary mastectomy.

## Case presentation

**Fig. 1:**
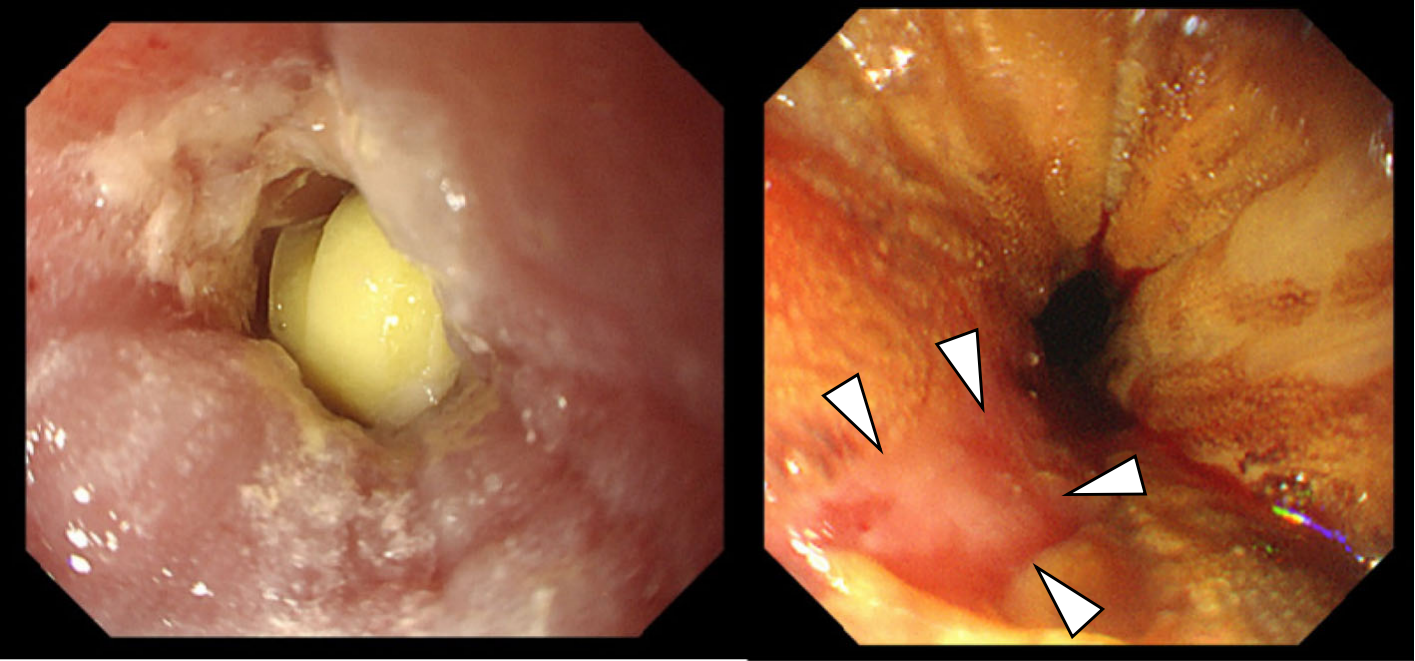
Upper gastrointestinal tract endoscopic examination findings. **a** Circumferential stenosis and lumen occluded by a tablet 30 cm from incisors are visible. **b** A 2-cm of band unstained by Lugol’s solution (arrows) was observed

**Fig. 2:**
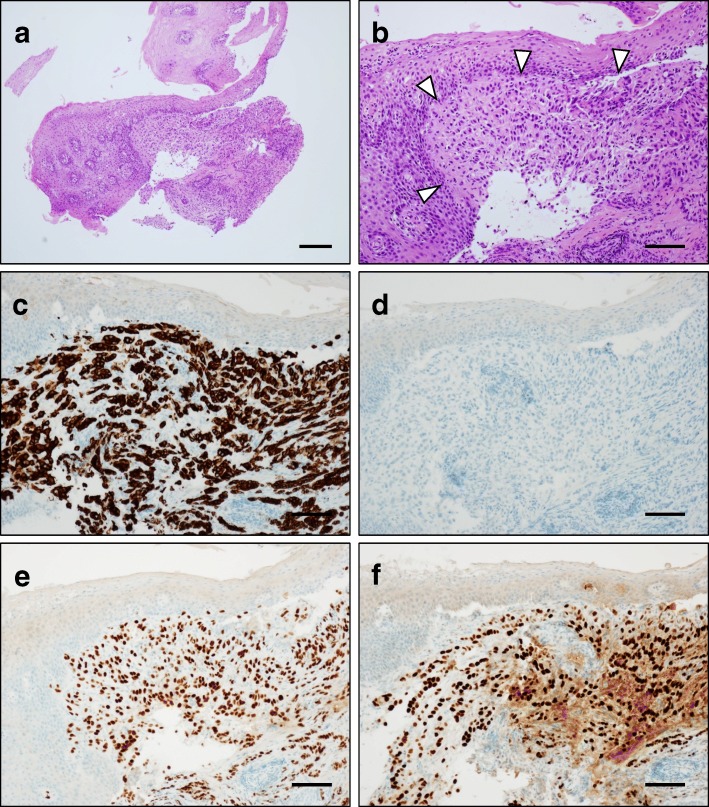
Histopathological findings of the esophageal tumor. Hematoxylin-eosin staining of biopsy specimens shows a subepithelial luminal structure and dysplastic cells covered with normal squamous epithelia (**a** low-power view, **b** high-power view). Immunostaining was positive for CK7 (**c**), negative for CK20 (**d**), positive for ER (**e**), and positive for PgR (**f**). Scale bars, 200 μm (**a**) and 100 μm (**b**-**f**)

**Fig. 3:**
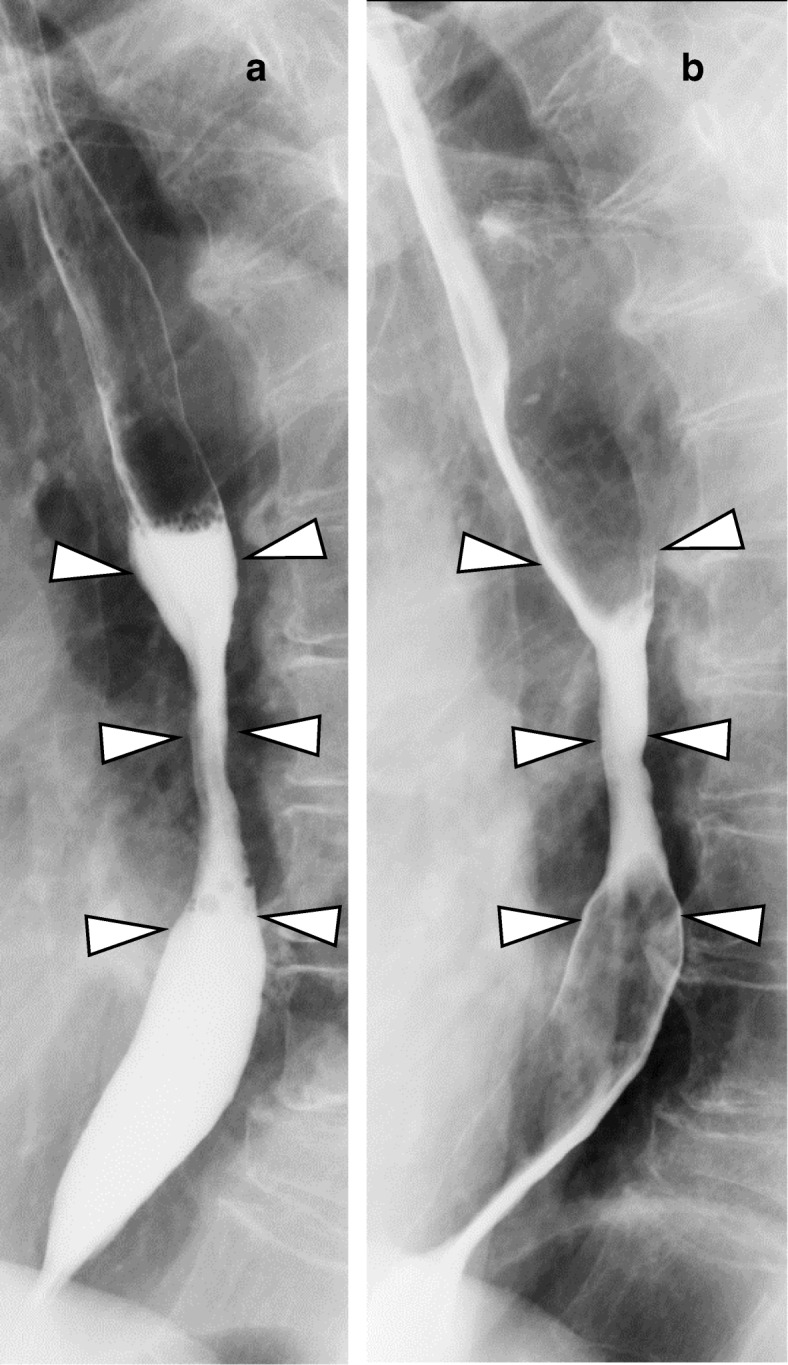
Esophagography findings. Esophagography showing a mid-esophageal stenosis of more than 5 cm in length and a minimal luminal diameter around 3 mm (**a**). After 3 months of endocrine therapy, the stenosis of the esophageal lumen is improved (**b**)

**Fig. 4:**
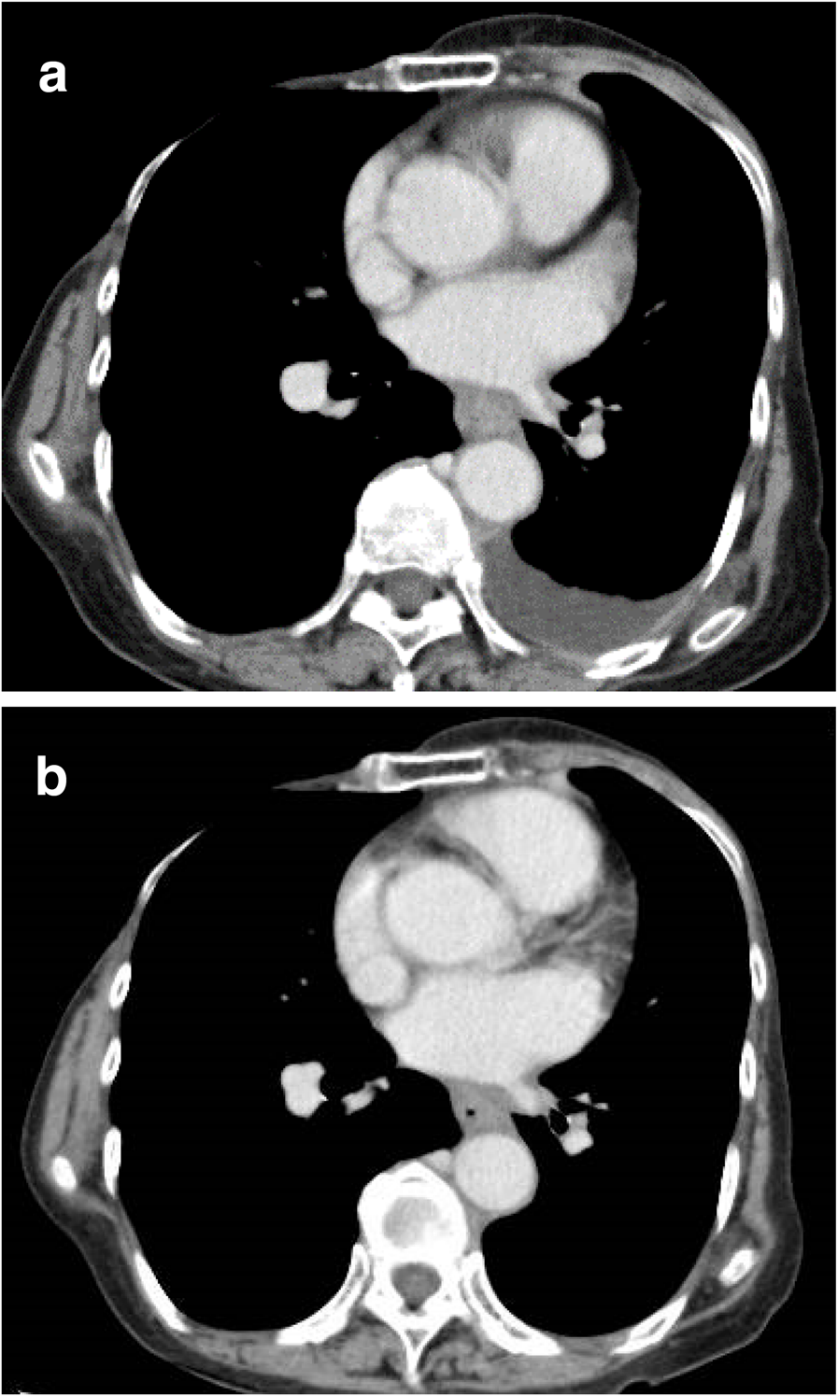
Thoracic enhanced CT findings. Thoracic enhanced CT showing wall thickening of mid-esophagus and left pleural effusion (**a**). After 3 months of endocrine therapy, the pleural effusion has disappeared (**b**)

A 77-year-old woman with a history of heterochronous bilateral breast cancer complained of dysphagia. At the age of 56, she had undergone a right radical mastectomy for right breast cancer. Histopathological examination revealed invasive ductal carcinoma, pT3N1M0 that was estrogen receptor (ER)- and progesterone receptor (PgR)-positive. The human epidermal growth factor receptor 2 (HER2) status was not assessed at that time. She had taken doxifluridine (5-DFUR) for 2 years and then tamoxifen for 5 years as adjuvant therapy. At the age of 73, a screening mammogram revealed a left breast mass and she was diagnosed with left breast cancer after examination. She underwent a second operation, a left modified radical mastectomy. The histopathological findings revealed a 12-mm apocrine carcinoma of the left breast without lymph node metastasis. The cancer was negative for the expression of ER, PgR, and HER2. The patient was administrated six courses of a combination therapy of cyclophosphamide, methotrexate, and 5- fluorouracil (CMF) as adjuvant therapy. After a 4-year absence, a regularly scheduled check-up revealed pleural effusion on her left side that increased in size at a 6-month re-examination. Pleuorocentesis performed to harvest the pleural effusion revealed pleural dissemination. Considering that the cell block obtained from the pleural effusion was ER-positive, she was diagnosed with a recurrence of her right breast cancer. She had a history of compressed fracture and was under treatment for osteoporosis; therefore, the patient was treated not with an aromatase inhibitor but with high-dose toremifene citrate. After initiation of therapy, she developed dysphagia. An upper gastrointestinal tract endoscopic examination revealed a whole circumferential stenosis 30 cm from her incisors and a 2-cm band unstained by Lugol’s solution. At this point, the lumen was occluded by a toremifene citrate tablet. Transoral endoscopy was unsuccessful; therefore, the tablet was pushed back into the stomach by small diameter endoscopy (Fig. 1). Hematoxylin-eosin staining of the biopsy specimen revealed a subepithelial luminal structure and dysplastic cells covered with normal squamous epithelia, and immunostaining was positive for CK7 and negative for CK20 (Fig. 2). An esophageal submucosal tumor originating in breast cancer was suspected based on the pathological findings. The specimen was positive for ER and PgR; therefore, the patient received a definitive diagnosis of esophageal metastasis of her right breast cancer 21 years after surgery. Esophagography revealed mid-esophageal stenosis more than 5 cm in length, with a minimal luminal diameter of around 3 mm (Fig. 3 a). Computed tomography (CT) also revealed wall thickening of the mid-esophagus, but there was no swelling of the lymph node around the mediastinal level (Fig. 4a). After 3 months of high-dose toremifene therapy, the pleural effusion had disappeared on CT (Fig. 4b), and her dysphagia was improved. Esophagography showed improvement of the esophageal lumen stenosis (Fig. 3b). High-dose toremifene was effective for 8 months, but the patient complained of dysphagia again and was diagnosed with progressive disease at the esophagus. She received 60 Gy of radiation at the middle esophagus and was administrated fulvestrant instead of high-dose toremifene. Fulvestrant has been effective for 16 months, up to now.

## Discussion

In an autopsy study of 1020 patients by Yanagawa et al. [1], primary lesions of esophageal metastasis were observed primarily in the stomach (308 cases, 30.2%); the lungs, trachea, and bronchus (291 cases, 28.5%); and hematopoietic bone marrow (87 cases, 8.5%); in contrast, only 22 cases (2.2%) originated from the breast. In another autopsy case report, Abrams et al. [2] reported a prevalence of esophageal metastasis from breast cancer of 4.2%. However, Borst and Ingold reported esophageal metastasis in only 0.4% of 2246 breast cancer patients followed for 18 years [3]. Asch et al. [4] also reported that 5.9% of autopsies of patients with breast cancer had esophageal metastasis but that only 0.59% had complained of dysphagia. In light of these reports, metastasis to the esophagus in breast cancer patient is relatively rare and the prevalence of symptoms differs from the frequency at which it is identified during autopsy.

According to the literature, 27 cases of esophageal metastasis from breast cancer including our case have been reported in the last 20 years [5–22]. A summary of these cases is shown in Table 1. The median age of diagnosis of esophageal metastasis was 68 years and most of the patients complained of dysphagia as a primary symptom (data not shown). Hormone receptor and HER2 expression were described in eight cases, all of which expressed hormone receptors. Esophageal metastases developed most commonly in the middle thoracic esophagus and extended up to 10 cm in length. The median time from the operation for breast cancer to the onset of esophageal metastasis was 11 years; interestingly, intervals more than 20 years were reported in four cases including ours [12, 13, 20].

**Table 1:** Esophageal metastasis from breast cancer for the past 20 years

Ref No.	Year	Age	Sex	Primary site		Location	Length (cm)	DFS (year)	Latent interval (year)	Initial biopsy pathology	Synchronous other metastasis site	Treatment						Prognosis (month)	Status
				ER/PgR	HER2							C	E	O	R	S	D		
5	1997	70	F	n/a	n/a	Mt	10	2	2	Benign	Local recurrence	○	○		○			36	Alive
6	1997	68	F	n/a	n/a	Mt	2	15	15	MBC	LN		○	○				12	Alive
7	1997	39	F	n/a	n/a	Lt, Ae	5	2	4	Benign	Lung, liver				○	○		4	Dead
8	1997	56	F	n/a	n/a	Lt	5	7	7	Benign	LN	○	○	○				48	Dead
9	1998	83	F	n/a	n/a	Ce	n/a	13	13	MBC	Liver		○				○	n/a	n/a
10	2001	73	F	n/a	n/a	Lt	7	9	9	MBC	n/a						○	12	Dead
10	2001	41	F	n/a	n/a	Mt	4	4	4	Benign	n/a	○				○	○	1	Dead
10	2001	77	F	n/a	n/a	Mt	n/a	3.5	3.5	Benign	n/a						○	18	Alive
10	2001	74	F	n/a	n/a	Mt	n/a	11	11	MBC	n/a					○	○	5	Dead
11	2002	55	F	n/a	n/a	Mt	n/a	11	11	Benign	None	○	○		○			36	Alive
12	2005	76	F	+	n/a	Ce	2	5	6	Benign	Bone	○	○			○		33	Alive
13	2005	72	F	n/a	n/a	Mt	n/a	23	23	Benign	None			○				12	Alive
13	2005	52	F	n/a	n/a	Lt	n/a	10	10	MBC	Pleura, lung, liver, adrenal gland, bone, brain		○			○	○	n/a	n/a
14	2005	68	F	n/a	n/a	Mt	n/a	23	23	Benign	None		○				○	14	Alive
15	2005	66	F	n/a	n/a	Ce	n/a	7	7	MBC	n/a	○			○			52	Dead
16	2006	67	F	n/a	n/a	EGJ	n/a	19	19	Benign	Lung		○	○				6	Alive
17	2009	70	F	+	n/a	Mt	3.5	16	16	MBC	None		○		○			30	Alive
18	2012	55	F	+	n/a	Ut, Mt	10	11	12	Undiagnosed	Local, LN	○						10	Alive
18	2012	61	F	+	–	Mt	7	6	8	MBC	Lung, pleura, LN, liver, brain	○						9	Alive
18	2012	64	F	+	–	Ut, Mt	n/a	4	9	Undiagnosed	Local, bone		○		○			6	Dead
18	2012	68	F	+	–	Mt	6	9	13	MBC	Bone, pleura	○						4	Alive
19	2013	61	F	n/a		Mt	n/a	15	15	MBC	None		○		○			n/a	n/a
1	2013	83	F	+	–	Mt	5	2	7	MBC	LN, lung	○				○		23	Dead
20	2014	79	F	n/a	n/a	Mt	3	15	26	MBC	None	○	○	○				96	Alive
21	2015	84	F	n/a	n/a	Lt	7	14	14	Benign	None	n/a						n/a	n/a
22	2015	62	F	n/a	n/a	Mt	5	13	13	Benign	None		○	○		○		60	Alive
Our	2018	77	F	+	n/a	Mt	5	21	21	MBC	Pleura		○		○			24	Alive

*ER* estrogen receptor, *PgR* Progesterone receptor, *HER2* human epidermal growth factor 2, *DFS* disease-free survival, *Ce* cervical esophagus, *Mt* middle thoracic esophagus, *Lt* lower thoracic esophagus, *Ae* abdominal esophagus, *EGJ* esophagogastric junction, *MBC* metastasis of breast cancer, *C* chemotherapy, *E* endocrine therapy, *O* operation, *R* radiation, *S* stenting, *D* dilation, *n/a* not applicable

The initial biopsy failed to definitively diagnose esophageal metastasis in almost half of the cases. This is because metastatic lesions are often located in the submucosa, and mucosal biopsy generally does not provide a sufficient amount of sample for definitive diagnosis. We did not use a special method for biopsy; however, we could collect a sufficient amount of sample, including submucosal tissue, for diagnosis of esophageal metastasis in this case. According to Matsumoto et al. [21], mediastinoscopy or surgery may be required for a definitive diagnosis; they also suggested endoscopic ultrasound-guided fine-needle biopsy as an alternative diagnostic tool.

The mechanism for how breast cancer spreads to the esophagus is unclear. There are two possible pathways for metastasis from the breast to the esophagus: lymphogenous metastasis of the paraesophageal lymph nodes via the parasternal lymph and mediastinum, and hematogenous metastasis in case of the absence of mediastinal lymph node swelling [23]. Indeed, 8 of 27 cases in the literature did not have any metastasis other than that of the esophagus, while 9 of 27 cases synchronously had loco-lesional recurrence, 9 of 27 cases had distant metastasis, and other metastasis sites were not reported in six of the 27 cases (Table 1). In our case, mediastinal lymph node swelling was not observed. However carcinomatous pleurisy originated from breast cancer occurs as a result of lymphangitis-type and subpleural lymphatic progress [24]. The patient had pleural dissemination; therefore, lymphogenous rather than hematogenous metastasis was suspected.

The treatment for esophageal metastasis also varies (Table 1). Twenty-one of 27 cases were administrated systemic therapy including chemotherapy and endocrine therapy; 14 of 27 cases received local treatment, including surgery and radiation therapy; and 11 of 27 cases were treated endoscopically. Goldberg et al. [25] reported that esophageal metastasis originating from breast cancer often occurred as a part of multiple-organ metastases, resulting in poor prognosis. In this case, pleural dissemination was diagnosed initially and systemic treatment was started; then, dysphagia was observed that led to the diagnosis of esophageal metastasis. On retrospective examination of a CT scan taken at the time she was diagnosed with pleural dissemination, we observed wall thickening of the mid-esophagus. Thus, we inferred that this esophageal metastasis was a part of organ metastasis. Systemic therapy is important for the management of multiple organ metastases. However, patients with only esophageal metastasis survived for more than 5 years after metastasis onset [20, 22]. Sato et al. [18] suggested that the esophagus could be a site of primary recurrence; thus, the possibility of esophageal metastasis should be considered during follow-up examinations to maintain a disease-free status. These cases may also result from lymphogenous metastases; thus, local control by surgery or radiation therapy may be effective.

Esophageal metastasis causes dysphagia and can seriously decrease patient quality of life but is usually not considered a prognostic factor for patients. Therefore, the treatment should be decided according to the severity of other metastatic sites and on the degree of the esophageal stenosis. Atkins et al. [26] reported that radiotherapy only or combined with chemotherapy were efficacious treatments for dysphagia caused by esophageal metastatic lesions. Other reports showed that endocrine monotherapy remarkably improved symptoms [14]. Surgical resection is usually not selected because systemic therapy takes priority for metastatic breast cancer. In the literature, three cases survived more than 20 months after surgical resection to esophageal metastasis. For cases with esophageal metastasis without other severe visceral metastases, surgical resection may provide good local control. However, surgical resection requires careful consideration because of its severe adverse events. Endoscopic dilation is not considered a standard therapy due to the risk of perforation. Sugauchi et al. [7] reported a very high rate of perforation; however, the recent use of self-expanding metallic stents may result in good outcomes.

In our case, endocrine therapy was started initially for pleural dissemination, and then, she was diagnosed with esophageal metastasis with dysphagia. After the patient developed resistance to the initial endocrine therapy, radiation therapy combined with secondary endocrine therapy was started, and it has been effective for more than 15 months, suggesting the importance of local control for esophageal metastasis.

## Conclusions

The prevalence of the symptoms of esophageal metastasis originating in breast cancer differs from its prevalence in autopsies; therefore, it is likely that there is a comparatively large number of breast cancer patients with latent esophageal metastasis. Metastatic lesions in the esophagus are often located in the submucosa; therefore, they may not be diagnosed definitively by single histopathological examination of mucosal biopsy specimens. Esophageal metastasis originating from breast cancer often occurs as a part of multiple organ metastases, and radiotherapy only or combined with chemotherapy are efficacious treatments for dysphagia. However, esophageal metastasis is not usually considered a prognostic factor. Therefore, treatment should be decided according to the severity of the other metastatic sites and the degree of esophageal stenosis.

## Abbreviations

5-DFUR: Doxifluridine
CMF: Cyclophosphamide, methotrexate, and 5-fluolouracil
CT: Computed tomography
ER: Estrogen receptor
HER2: Human epidermal growth factor 2
PgR: Progesterone receptor

## References

[1] Yanagawa T, Yoshida T, Kimura Y, Watanabe A, Imaoka S (2013). Esophageal metastasis of breast cancer with esophagobronchial fistula —a case report—(in Japanese with English abstract). J Jpn Surg Assoc.

[2] Abrams HL, Spiro R, Goldstein N (1950). Metastases in carcinoma; analysis of 1000 autopsied cases. Cancer.

[3] Borst MJ, Ingold JA (1993). Metastatic patterns of invasive lobular versus invasive ductal carcinoma of the breast. Surgery.

[4] Asch MJ, Wiedel PD, Habif DV (1968). Gastrointestinal metastases from carcinoma of the breast. Autopsy study and 18 cases requiring operative intervention. Arch Surg.

[5] Kono K, Murakami M, Okamoto Y, Yoden E, Kobayashi K, Kuroda Y (1997). Experience of radiation therapy for esophageal metastasis from breast cancer; a case report (in Japanese with English abstract). Jpn J Clin Radiol.

[6] Fujii K, Nakanishi Y, Ochiai A, Tsuda H, Yamaguchi H, Tachimori Y (1997). Solitary esophageal metastasis of breast cancer with 15 years’ latency: a case report and review of the literature. Pathol Int.

[7] Sugauchi F, Yamaguchi H, Kondou H, Shirao K, Ono H, Iwabuchi M (1997). A case of successful treatment with a self-expanding metal stent for esophageal stenosis caused by metastasis of breast cancer (in Japanese with English abstract). Gastroenterol Endosc.

[8] Mizobuchi S, Tachimori Y, Kato H, Watanabe H, Nakanishi Y, Ochiai A (1997). Metastatic esophageal tumors from distant primary lesions: report of three esophagectomies and study of 1835 autopsy cases. Jpn J Clin Oncol.

[9] Wu CM, Hruban RH, Fishman EK (1998). Breast carcinoma metastatic to the esophagus. CT findings with pathologic correlation. Clin Imaging.

[10] Simchuk EJ, Low DE (2001). Direct esophageal metastasis from a distant primary tumor is a submucosal process: a review of six cases. Dis Esophagus.

[11] Erman M, Karaoğlu A, Oksüzoğlu B, Aydingöz U, Ayhan A, Güler N (2002). Solitary esophageal metastasis of breast cancer after 11 years: a case report. Med Oncol.

[12] Tachibana A, Fukuda M, Ui Y, Yamakawa T (2005). Esophageal metastasis of breast cancer treated with self expandable metallic stent of a case (in Japanese with English abstract). Gastroenterol Endosc.

[13] Koike M, Akiyama S, Kodera Y, Nakao A (2005). Breast carcinoma metastasis to the esophagus: report of two cases. Hepato-Gastroenterology.

[14] Sunada F, Yamamoto H, Kita H, Hanatsuka K, Ajibe H, Masuda M (2005). A case of esophageal stricture due to metastatic breast cancer diagnosed by endoscopic mucosal resection. Jpn J Clin Oncol.

[15] Shiraishi K, Wakita A, Ishigami I, Tomita K, Yamamoto A, Nakayama T (2005). Palliative home care of a breast cancer patient with esophageal metastasis after the operation of gastric fistula and tracheotomy--a case study (in Japanese with English abstract). Jpn J Cancer Chemother.

[16] Anaya DA, Yu M, Karmy-Jones R (2006). Esophageal perforation in a patient with metastatic breast cancer to esophagus. Ann Thorac Surg.

[17] Wada Y, Harada N, Ohara K, Kawata H, Iwasaki H, Kawamura Y (2009). Esophageal metastasis of breast carcinoma. Breast Cancer.

[18] Sato Y, Horiguchi H, Yoshida M, Nakamura T, Sagawa T, Shintani N (2012). Four cases of esophageal metastasis from breast cancer (in Japanese with English abstract). J Jpn Society Gastoroenterol.

[19] Talanow R, Vieweg H, Andresen R (2013). Solitary breast cancer metastasis to the esophagus – a multimodal diagnostic approach. Z Gastroenterol.

[20] Kawabata R, Kimura Y, Kawase T, Kamigaki S, Yamamura J, Nakamura Y (2014). Long-term survival of a patient with esophageal metastasis from breast cancer treated with esophagectomy (in Japanese with English abstract). Jpn J Cancer Chemother.

[21] Matsumoto Y, Matsukawa H, Seno H, Ono S (2015). Education and imaging. Gastrointestinal: breast cancer metastasis to the esophagus diagnosed using endoscopic ultrasound-guided fine-needle aspiration. J Gastroenterol Hepatol.

[22] Wilson MA, Shah N, O'Donnell ME, Jaroszewski DE (2015). An unusual presentation of esophageal metastasis from breast cancer. J Thorac Cardiovasc Surg.

[23] Boccardo F, Merlano M, Canobbio L, Rosso R, Aste H (1982). Esophageal involvement in breast cancer. Report of six cases. Tumori.

[24] Yoshikawa K, Iwai K, Wada M, Sugita G, Kawabata Y (1981). Clinicopathological studies on cancerous pleurisy, with emphasis on extra-pulmonary malignancy. Jpn J Lung Cancer.

[25] Goldberg RI, Rams H, Stone B, Barkin JS (1987). Dysphagia as the presenting symptom of recurrent breast carcinoma. Cancer.

[26] Atkins JP (1966). Metastatic carcinoma to the esophagus. Endoscopic considerations with special reference to carcinoma of the breast. Ann Otol Rhinol Laryngol.

